# Advantages of Hyperbaric Oxygen Treatment for Type 2 Diabetes Mellitus in Mice: Involvement of Leptin

**DOI:** 10.1155/jdr/3808140

**Published:** 2025-09-28

**Authors:** Zhounan Zhu, Bin Zhang, Wanyin Wang, Hongjie Yi, Chenggang Zheng, Qinghe Tang

**Affiliations:** ^1^Department of Hepatobiliary Pancreatic Surgery, Shanghai East Hospital, School of Medicine, Tongji University, Shanghai, China; ^2^Department of Hyperbaric Oxygen Treatment, First Affiliated Hospital of Naval Military Medical University, Shanghai, China; ^3^Clinical Research Institute, Third Affiliated Hospital of Naval Military Medical University, Shanghai, China

**Keywords:** blood glucose, hyperbaric oxygen treatment, leptin, liver steatosis, Type 2 diabetes mellitus

## Abstract

**Background:** Hyperbaric oxygen treatment (HBOT) is clinically used to improve oxygen supply to hypoperfused tissues under certain conditions. HBOT can decrease the incidence of autoimmune diabetes in nonobese diabetic mice by reducing apoptosis and increasing *β*-cell proliferation. HBOT ameliorates glucose tolerance in Type 2 diabetes (T2D) mellitus patients, but the underlying mechanism needs further investigation.

**Methods:** We used a diet-induced T2D mouse model and a genetic mouse model (ob/ob mice) to evaluate the effects of HBOT on serum glucose levels in mice. The body weights and blood glucose levels of the mice were measured weekly. An oral glucose tolerance test (OGTT) was performed 12 weeks after the start of the experiment. All the mice were euthanized, and the serum and liver tissues were collected to test the total cholesterol, triglycerides, alanine aminotransferase, aspartate aminotransferase, malondialdehyde, and antioxidant enzymes.

**Results:** Our results demonstrated that HBOT can delay/attenuate the onset of diet-associated T2D in wild-type mice. However, HBOT had no significant effects on blood glucose or T2D incidence in ob/ob mice. Furthermore, we found that HBOT improved glucose tolerance and liver steatosis in diet-induced T2D model mice but not in ob/ob mice. Our results indicated that the effects of HBOT on T2D were dependent at least partly on the presence of leptin.

**Conclusion:** Our study offers a rationale for using serum leptin as a predictor of clinical outcomes of HBOT and elucidates possible reasons why many patients may experience HBOT failure.

## 1. Introduction

Type 2 diabetes (T2D) mellitus is a rapidly increasing global metabolic disease. The prevalence of T2D has risen dramatically over the past few decades [1]. The International Diabetes Federation (IDF) estimated that the number of T2DM cases will increase to 642 million by 2040 [1]. T2D leads to uncontrolled glucose and lipid metabolism accompanied by insulin resistance and related complications, including hyperglycemia, hyperlipidemia, hypertension, and cardiovascular diseases, which are the leading causes of death in T2D patients [2]. All these factors impose a great burden on public health. Prevention or attenuation of T2D will contribute to reducing both social and financial burdens.

Medical hyperbaric oxygen treatment (HBOT) is a treatment regimen in which patients breathe 100% oxygen at a pressure of > 1 atmosphere absolute (ATA) for 1–2 h per session [3]. HBOT has been demonstrated to have multiple beneficial effects; it is clinically used to improve the oxygen supply to hypoperfused tissues under certain conditions, such as carbon monoxide exposure and ischemic events. HBOT was reported to have anti-inflammatory effects and to assist in the treatment of diseases [3–6]. Recently, HBOT has been demonstrated to improve early bone regeneration in Type 1 diabetic rats [7]. HBOT can also reduce the incidence of autoimmune diabetes in NOD (no obesity diabetes) mice by increasing the number of resting T cells and reducing the activation of DCs (dendritic cells) while preserving the *β*-cell mass resulting from decreased apoptosis and increased proliferation [8]. HBOT has been reported to ameliorate glucose intolerance in T2D patients [9]. However, the mechanism by which HBOT ameliorates serum glucose remains poorly defined.

In the present study, we used a diet-induced mouse T2D model and a genetic mouse model (ob/ob mice) to evaluate the effects of HBOT on serum glucose levels in mice. We observed the levels of serum insulin, leptin, and resistin during HBOT.

## 2. Methods

### 2.1. Animals

The studies were approved by the animal care and use committee of the Naval Military Medical University. ICR (Institute of Cancer Research) mice, which are commonly used in cancer research due to their robust health and high reproducibility in various experiments, and ob/ob mice, which are mice that are genetically predisposed to obesity and exhibit characteristics such as high body fat, resistance to leptin, and a tendency to become overweight, with a maximum weight of up to 60 g, were used in this study. These mice are infertile and typically exhibit simple obesity without branuria. C57 mice were obtained from Shilaike Experiment Animal Limited Company (Shanghai, China). All the mice were housed in virus antibody-free microisolation cages and exposed to a 12-h dark/light cycle with ad libitum access to water. The food and food quantities were provided as described in the experimental protocols.

### 2.2. HBOT

**Figure 1:**
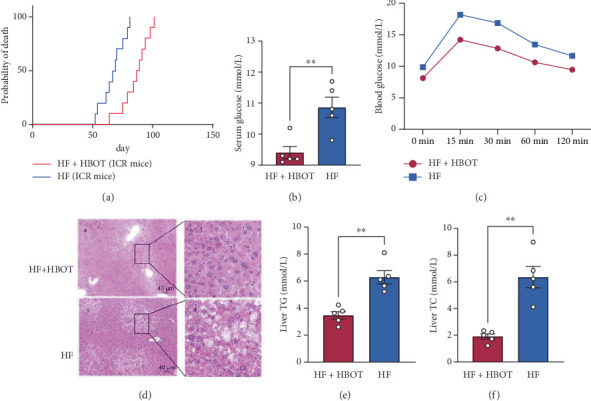
Hyperbaric oxygen treatment (HBOT) and its effects on Type 2 diabetes (T2D) incidence and metabolic parameters. (a) Progress curve of T2D incidence in male Institute of Cancer Research (ICR) mice fed a high-fat and high-glucose diet (HF) for 20 weeks, with or without HBOT administration. Blood glucose was monitored, and a nonfasting glycemic value of 16.7 mmol/L on two consecutive readings was defined as T2D (*n* = 10). (b) Serum glucose levels of male C57 mice fed an HF for 14 weeks with or without HBOT administration. (c) Oral glucose tolerance test (OGTT) curve of male C57 mice fed an HF for 12 weeks with or without HBOT administration. (d) Representative histological images of liver steatosis in male C57 mice fed an HF for 12 weeks with or without HBOT administration ((a, c) 100× and (b, d) 400×). (e) Liver triglyceride (TG) levels in the HF and HF + HBOT groups. (f) Liver total cholesterol (TC) levels in the HF and HF + HBOT groups. Data are presented as mean ± SEM (⁣^∗∗^*p* < 0.01, *n* = 5).

HBOT was carried out in a hyperbaric chamber for animal experiments once a day, 5 times per week, from Monday to Friday. The treatment session is shown in Figure 1a. Treatment started at 8:30 a.m. every day. The pressure was increased from 1 to 2.5 ATA over a 5-min period, followed by 60 min of continuous exposure to either 100% oxygen (HBOT) or a 21% oxygen air mixture (to achieve normoxia, i.e., 21% oxygen tension in tissues) at a pressure of 2.5 ATA. At the end, the pressure in the chamber was slowly reduced to 1 ATA over a 5-min period before the chamber was opened.

### 2.3. Experimental Protocols

In the first experiment, 40 male ICR mice weighing 22–25 g were randomly divided into four groups: the normal diet (ND) group; the HBOT-normal diet (ND + HBOT) group; the high-glucose and high-fat diet (HF) group; and the HF with HBOT (HF + HBOT) group. All the mice had ad libitum access to food. A high-fat diet purchased from Research Diets (60% fat, D12492; Research Diets) was given to the mice in the HF and HF + HBOT groups. On the first day of diet administration, the mice in the ND + HBOT and HF + HBOT groups were exposed to 100% oxygen continuously for 60 min, and the mice in the ND and HF groups were exposed to 21% oxygen continuously for 60 min. The body weights and blood glucose levels of the mice were measured weekly. The food intake of the mice in all the groups was measured three times per week on Monday, Wednesday, and Friday. Diabetes was defined as fasting glycemic values of 11.1 mmol/L or nonfasting glycemic values of 16.7 mmol/L on two consecutive readings. The experiment ended when all the mice in the HF and HF + HBOT groups were diagnosed with diabetes.

In the second experiment, 15 male C57 mice were grouped and treated as described in the first experiment. C57 mice were used to minimize the individual variability associated with the ICR strain mice. Our preliminary experiment revealed that during the modeling period, mice fed a ND did not exhibit any significant differences in blood glucose or liver function, regardless of whether they were treated with HBOT. Therefore, the ND + HBOT group was omitted from this experiment. The three groups were as follows: the normal diet group without HBOT (ND group), the high-fat and high-glucose diet group with HBOT (HF + HBOT group), and the high-fat and high-glucose diet group without HBOT (HF group), with five mice in each group. The mice in all the groups were subjected to an OGTT (oral glucose tolerance test). The experiment ended 2 weeks after the OGTT. All the mice were euthanized, and the serum and liver tissue were collected for experimental use.

In the third experiment, 10 C57 male ob/ob mice were equally divided into two groups: a high-glucose and high-fat diet group without HBOT (HF ob/ob group) and a high-glucose and high-fat diet group with HBOT (HF + HBOT ob/ob group). The ob/ob mice were subjected to restricted feeding, and the daily food supply was limited to 4.5 ± 0.5 g per mouse, according to the average intake observed in the C57 mice from the second experiment. The mice were subjected to an OGTT 12 weeks after the start of the experiment. The mice in the ob-test group were exposed to 100% oxygen continuously for 60 min; at the same time, the mice in the ob-con group were exposed to 21% oxygen continuously for 60 min. The experiment ended 2 weeks after the OGTT. All the mice were euthanized, and the serum and liver tissues were collected for experimental use.

### 2.4. Blood Glucose Measurements

The blood glucose levels of the animals in all groups were measured weekly by a glucometer (Accu-check, Roche Diagnostics GmbH, Mannheim, Germany) via blood collected from the tail vein. To ensure consistency, the mice were fasted for 6 h from 10:00 a.m. every Saturday, and blood glucose was measured at 4:00 p.m. on the same day.

### 2.5. OGTT

The OGTT was performed as previously described [10]. Briefly, at the 12th week after the start of the experiment, the mice were fasted for 16 hr, after which each mouse received a glucose solution prepared with sterile water (100 mg/mL) (Sigma Aldrich) by oral gavage (2 g/kg). Blood glucose levels were measured via blood samples collected from the tail vein before oral gavage (min 0) and at 15, 30, 60, and 120 min after gavage, as described above.

### 2.6. Histopathological Examination

Mouse liver tissues were fixed in formalin and embedded in paraffin. Then, 5-μm-thick sections were cut and examined. Hematoxylin–eosin staining was performed to assess hepatic morphology. Oil Red O staining was used to assess hepatic fat deposition.

### 2.7. Cytokine and Diabetes-Related Factor Detection

Mouse serum was assayed for diabetes-related factors, including leptin, resistin, GIL-1, and ghrelin, using the Bio-Plex Pro Mouse Cytokine/Diabetes Factor Multiplex Assay on a BioPlex instrument (Bio-Rad) according to the protocol.

### 2.8. Measurement of Malondialdehyde (MDA)

Oxidative stress was evaluated by measuring the levels of thiobarbituric acid-reactive substances (TBARS) in liver homogenates, where the liver samples were homogenized in chilled 1.15% KCl buffer following established protocols, with MDA used as the reference standard for quantifying TBARS, as described by He et al. and Nakade et al [10, 11].

### 2.9. Real-Time Polymerase Chain Reaction of Liver Tissue RNA

Frozen liver samples were first homogenized with TRIzol reagent (Life Technologies, Tokyo, Japan) to lyse the tissue, and RNA was subsequently extracted using the RNeasy Mini Kit (Qiagen, Tokyo, Japan). The isolated RNA was then resuspended in 20 *μ*L of RNase-free water, and its concentration was quantified using spectrophotometry at OD 260 and confirmed by low-mass gel electrophoresis (Invitrogen, Tokyo, Japan). For cDNA synthesis, the High-Capacity cDNA Reverse Transcription Kit (Applied Biosystems, Foster City, CA) was used following the manufacturer's instructions. Real-time quantitative PCR was performed with SYBR Green on the ABI StepOne Sequence Detection System (Applied Biosystems).

### 2.10. Statistical Analysis

All the statistical analyses were performed using GraphPad Prism 5.0. The data are expressed as the means ± SEMs. Student's *t* test was used for comparisons between two groups. One-way ANOVA followed by the Kruskal–Wallis test was used for multiple group comparisons. A *p* value < 0.05 was considered statistically significant.

## 3. Results

### 3.1. HBOT Attenuated or Delayed Diet-Induced T2D in Mice

To observe the effects of HBOT on the incidence of T2D in mice, the first experiment was performed in ICR mice. On Day 50 (Week 7), the body weights of the mice in the HF and HF + HBOT groups were 43.68 ± 2.18 g and 43.68 ± 2.17 g, respectively, which were significantly greater than those in the ND (35.67 ± 2.11 g) and ND + HBOT (35.67 ± 2.06 g) groups (*p* < 0.001). The results showed that a high-fat diet led to an obvious increase in mouse body weight compared with that of mice fed a normal chow diet. Along with increased body weight, mouse blood glucose increased, and T2D developed after 50 days of HF feeding (Figure 1a). HBOT significantly attenuated or delayed the onset of diabetes induced by HF (Figure 1a). The second experiment was performed in C57 mice. The body weights of C57 mice at week 12 in the HF and HF + HBOT groups were 36.35 ± 4.73 g and 36.87 ± 4.56 g, respectively, which were significantly greater than those in the ND group (29.45 ± 1.39 g) (*p* = 0.03 and *p* = 0.02, respectively). The blood glucose levels of the HBOT-treated mice were significantly lower than those of the HF-fed mice (Figure 1b). By performing an OGTT, we found that HBOT improved glucose tolerance (Figure 1c). The histomorphology results also revealed that HBOT attenuated HF-induced liver steatosis (Figure 1d). The liver triglyceride (TG) and total cholesterol (TC) levels were measured, and the results revealed that HBOT significantly decreased the total fat content in the livers of the mice (Figure 1e,f).

### 3.2. HBOT Affected Serum Leptin and Glucagon-Like Peptide-1 (GLP-1) Levels in C57 Mice

**Figure 2:**
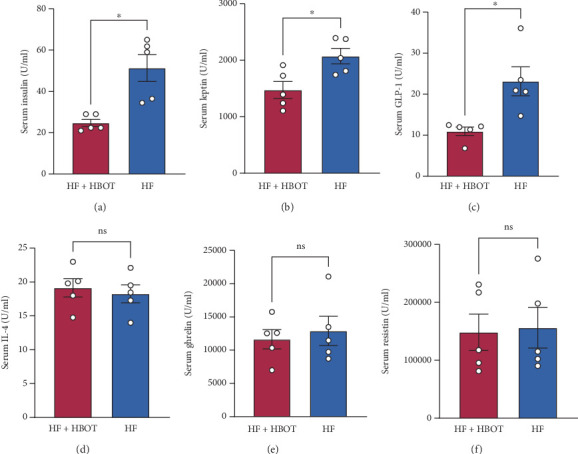
Hyperbaric oxygen treatment (HBOT) effects on serum leptin, glucagon-like peptide-1 (GLP-1), insulin, interleukin-4 (IL-4), ghrelin, and resistin levels in C57 mice. To investigate potential mechanisms by which HBOT attenuates or delays Type 2 diabetes (T2D), serum insulin, inflammation-related cytokines, and diabetes-related factors in C57 mice were measured using a Bio-Plex Pro Mouse Cytokine/Diabetes Factor Multiplex Assay Kit. Panels show serum levels of (a) leptin, (b) GLP-1, (c) insulin, (d) IL-4, (e) ghrelin, and (f) resistin in C57 mice fed a high-fat and high-glucose diet (HF) with or without HBOT administration (⁣^∗^*p* < 0.05; ns, not significant; *n* = 5).

To further investigate the possible mechanisms of HBOT, we tested cytokines and factors involved in diabetes modulation in the serum of experimental C57 mice. We found that HBOT decreased the serum insulin, leptin, and GLP-1 levels in the mice (Figures 2a, 2b, and 2c), but HBOT had no obvious effect on the serum interleukin-4 (IL-4), resistin, or ghrelin levels (Figures 2d, 2e, and 2f).

### 3.3. The Advantage of HBOT in the Treatment of Diabetes Is Dependent on the Presence of Leptin: Evidence From Ob/Ob Mice

**Figure 3:**
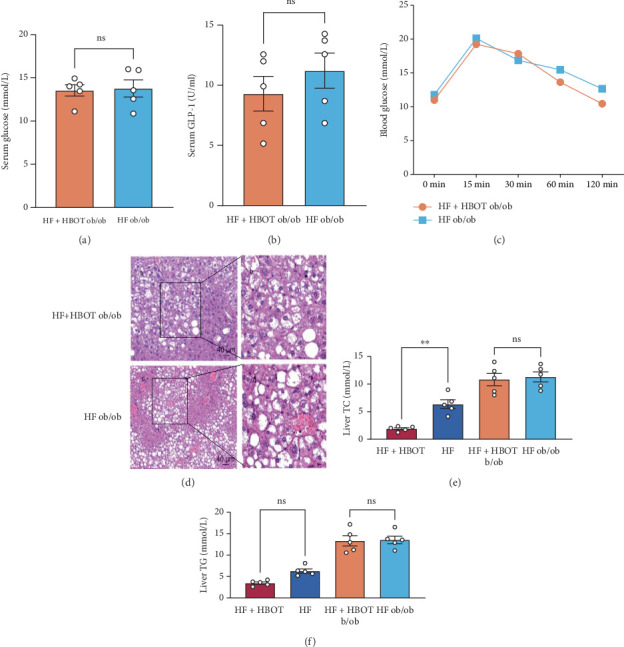
Serum metabolic parameters and liver histology in ob/ob mice with or without hyperbaric oxygen treatment (HBOT). Male ob/ob mice were used to test whether leptin was involved in the mechanism by which HBOT attenuated or delayed the onset of T2D. (a) Serum glucose levels of male ob/ob mice fed a high-glucose and high-fat diet for 14 weeks with or without HBOT administration. (b) Serum GLP-1 levels in male ob/ob mice fed a high-glucose and high-fat diet for 14 weeks with or without HBOT administration. (c) Oral glucose tolerance test (OGTT) curve of male ob/ob mice fed normal food for 14 weeks with or without HBOT administration. (d) Representative histomorphology image of liver steatosis in male ob/ob mice fed normal food for 14 weeks with or without HBOT administration ((a, c) 100× and (b, d) 400×). (e) Liver total cholesterol (TC) levels. (f) Liver triglyceride (TG) levels (⁣^∗∗^*p* < 0.01; ns, not significant; *n* = 5).

Based on the results above, we evaluated whether the beneficial effects of HBOT in T2D are dependent on leptin. To test this hypothesis, we examined the effects of HBOT on ob/ob mice, which are considered paradigmatic of nutritional obesity and develop hepatic steatosis and T2D due to a deficiency of the satiety hormone leptin. At Week 12, the average body weight was 52.15 ± 2.31 g in the HF ob/ob group and 52.79 ± 2.81 g in the HF + HBOT ob/ob group (*p* = 0.72), indicating that HBOT did not significantly influence body weight in leptin-deficient mice under limited restricted feeding conditions. Our results showed that HBOT had no influence on the serum levels of leptin and GLP-1 (Figure 3a,b) in ob/ob mice. It did not significantly affect glucose tolerance or liver steatosis, as shown by the results of the OGTT and H&E assays (Figure 3c,d). Although HBOT decreased liver TG and TC levels in HF-induced wild-type T2D mice (Figure 3e,f), it did not attenuate fat content in leptin-deficient mice.

**Figure 4:**
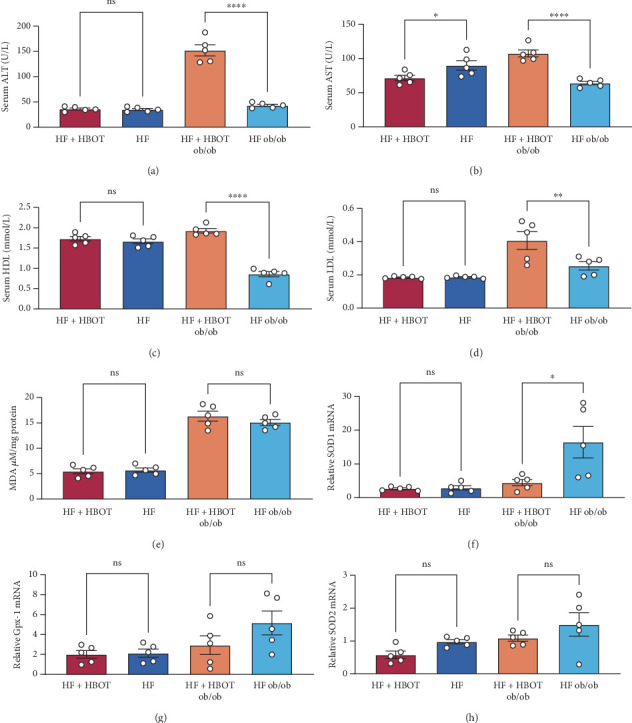
Serum biochemical indices and hepatic oxidative stress markers in mice with or without hyperbaric oxygen treatment (HBOT). (a) Serum alanine aminotransferase (ALT) levels. (b) Serum aspartate aminotransferase (AST) levels. (c) Serum high-density lipoprotein cholesterol (HDL) levels. (d) Serum low-density lipoprotein cholesterol (LDL) levels. (e) Hepatic malondialdehyde (MDA) levels. (f) Superoxide dismutase 1 (SOD1) mRNA expression. (g) Glutathione peroxidase 1 (Gpx-1) mRNA expression. (h) Hepatic superoxide dismutase 2 (SOD2) mRNA expression (⁣^∗^*p* < 0.05, ⁣^∗∗^*p* < 0.01, and ⁣^∗∗∗∗^*p* < 0.0001; ns, not significant; *n* = 5).

To our surprise, the serum AST and ALT levels in the HF + HBOT ob/ob group markedly increased compared with the HF ob/ob group (Figure 4a,b), indicating that HBOT induced hepatic injury in ob/ob mice. Consistently, the serum high-density lipoprotein (HDL) cholesterol and low-density lipoprotein (LDL) cholesterol levels in the HF + HBOT ob/ob group were much higher than those in the HF ob/ob group, indicating that HBOT impaired lipid metabolism in the HF ob/ob group (Figure 4c,d). We measured the MDA content in liver tissues (Figure 4e) and the relative mRNA levels of superoxide dismutase 1 (SOD1), glutathione peroxidase 1 (Gpx-1), and superoxide dismutase 2 (SOD2) in the liver (Figure 4f, 4g, and 4h) by real-time PCR. The results showed that HBOT had no significant effects on these indices, suggesting that HBOT did not induce oxidative stress.

## 4. Discussion

In the present study, we demonstrated that HBOT attenuated or delayed T2D. Furthermore, we investigated the possible mechanism by which HBOT reduces the incidence of T2D in HF-induced mice. Our results revealed decreased serum glucose, leptin, and GLP-1 and improved glucose tolerance in diet-induced model mice. HBOT had no significant effects on serum glucose or GLP-1 in ob/ob mice. These results indicate that the ability of HBOT to reduce diet-induced diabetes is dependent on the presence of leptin.

The advantages of HBOT for the treatment of diabetes, especially diabetic foot ulcers, have been reported. In humans, HBOT improved peripheral insulin sensitivity [4] and health-related quality of life in patients with diabetes and chronic foot ulcers [6]. In mice, HBOT delayed cataract development and progression in those with T2D [5]. Although HBO treatment is becoming more common, its mechanism is still not fully understood.

Obesity is widely accepted as a strong risk factor for the development of T2D, and obesity is considered to result from a passive imbalance of food intake and energy expenditure. With excessive nutrient intake, excess calories are stored in the body, especially in adipose tissue, resulting in obesity. Obesity plays an important role in the pathogenesis of T2DM. A high-glucose and high-fat diet is a widely accepted method to induce T2D in mice. We used a diet-induced mouse model to observe the effects of HBOT on diet-induced T2D. Our results revealed that the body weights of high-glucose and high-fat diet-fed mice increased faster than those of ND-fed mice. After 16 weeks of feeding, the body weights of the high-glucose and high-fat diet-fed mice were significantly greater than those of the ND-fed mice. HBOT had no effect on body weight in mice fed a high-glucose and high-fat diet or a ND. However, in mice fed a high-glucose and high-fat diet, fasting serum levels were lower in those treated with HBOT compared with non-HBOT-treated mice. The results of the glucose tolerance test also demonstrated that HBOT improved abnormal glucose tolerance.

It is well known that insulin, a hormone produced by the pancreas, is one of the main regulators of glucose concentrations in the blood. Insulin resistance serves as a key link between obesity and T2D [12]. To examine the possible mechanism by which HBOT modulates glucose in the blood, we observed whether HBOT could affect insulin levels in the blood. Using enzyme-linked immunosorbent assay (ELISA), we found a significant difference in serum insulin levels between mice treated with or without HBOT after approximately 12 weeks. HBOT preserves *β*-cell mass resulting from decreased apoptosis and increased proliferation in nonobese diabetic mice [13]. Under our experimental conditions, we found no significant effects of HBOT on *β*-cell mass or insulin mRNA. These results indicated that the effects of HBOT might indirectly affect mouse pancreas insulin expression or enhance insulin sensitivity. Whether HBOT affects insulin secretion requires further research.

To further research the possible mechanisms of HBOT, we tested several other factors that have been reported to be related to T2D, including resistin, leptin, ghrelin, and GLP-1. Our results revealed that the serum leptin and GLP-1 levels increased significantly in mice fed a high-glucose and high-fat diet. HBOT administration significantly attenuated the increase in serum leptin and GLP-1 in high-glucose and high-fat diet-fed mice. These results indicated that the modulation of leptin and GLP-1 may be involved in the mechanism of HBOT against T2D.

Next, we observed the effects of HBOT administration on ob/ob mice. We found that after 16 weeks of HBOT administration, there was no effect on mouse body weight, food intake, or serum glucose, insulin, or GLP-1 levels. However, compared with non-HBOT-treated mice, serum resistin and ghrelin increased significantly in mice that received HBOT. The mechanism by which HBOT stimulates the increase of serum resistin and ghrelin in ob/ob mice is currently under investigation.

HBOT has been reported to have disadvantages in mice with obesity, hyperlipidemia, and steatohepatitis, including the potential to cause organ damage by increasing oxidative stress [13]. Therefore, we investigated oxidative stress by MDA and the mRNA levels of antioxidant enzymes (SOD and GPx) in mouse liver tissue. Our results revealed that there were no significant differences in the MDA or mRNA levels of SOD or GPx in the livers of HF mice treated with or without HBOT. The same results were also obtained in mice fed high-glucose and high-fat diets with or without HBOT. These results indicated that HBOT did not trigger oxidative stress.

Because of the complicated modulation of energy metabolism in vivo, we did not further investigate the mechanism by which HBOT decreased leptin expression. Additionally, we did not further investigate the significance or mechanism by which HBOT altered the expression levels of IL-4, ghrelin, and resistin. These are interesting and significant issues to be addressed. We plan to focus on these problems in our ongoing work.

## 5. Conclusion

In the present study, we first demonstrated that HBOT improved glucose tolerance status, attenuated liver steatosis, and attenuated or delayed high-glucose and high-fat diet-induced T2D in mice in a leptin-dependent manner. Based on these results, serum leptin may serve as a useful biomarker to stratify T2D patients who may benefit from hyperbaric oxygen therapy in clinical practice.

## Data Availability

The data that support the findings of this study are available from the corresponding author upon reasonable request.
